# Protocol for the New Medicine Service Study: a randomized controlled trial and economic evaluation with qualitative appraisal comparing the effectiveness and cost effectiveness of the New Medicine Service in community pharmacies in England

**DOI:** 10.1186/1745-6215-14-411

**Published:** 2013-12-01

**Authors:** Matthew Boyd, Justin Waring, Nick Barber, Rajnikant Mehta, Antony Chuter, Anthony J Avery, Nde-Eshimuni Salema, James Davies, Asam Latif, Lukasz Tanajewski, Rachel A Elliott

**Affiliations:** 1 Division for Social Research in Medicines and Health, The School of Pharmacy, University of Nottingham, University Park, Nottingham NG7 2RD, UK; 2 Center for Health Innovation, Leadership & Learning, Nottingham University Business School, Jubilee Campus, University of Nottingham, Nottingham NG8 2BB, UK; 3 Department of Practice and Policy, UCL School of Pharmacy, 29-39 Brunswick Square, London WC1N 1AX, UK; 4 Trent Research Design Service, Division of Primary Care, Tower Building, University Park, Nottingham NG7 2RD, UK; 5 68 Brighton Cottages, Copyhold Lane, Lindfield, Haywards Heath RH16 1XT, UK; 6 Division of Primary Care, The Medical School, Queen’s Medical Center, Nottingham NG7 2UH, UK

## Abstract

**Background:**

Medication non-adherence is considered an important cause of morbidity and mortality in primary care. This study aims to determine the effectiveness, cost effectiveness and acceptability of a complex intervention delivered by community pharmacists, the New Medicine Service (NMS), compared with current practice in reducing non-adherence to, and problems with, newly prescribed medicines for chronic conditions.

**Methods/design:**

Research subject group: patients aged 14 years and above presenting in a community pharmacy for a newly prescribed medicine for asthma/chronic obstructive pulmonary disease (COPD); hypertension; type 2 diabetes or anticoagulant/antiplatelet agents in two geographical regions in England.

Design: parallel group patient-level pragmatic randomized controlled trial.

Interventions: patients randomized to either: (i) current practice; or (ii) NMS intervention comprising pharmacist-delivered support for a newly prescribed medicine.

Primary outcomes: proportion of adherent patients at six, ten and 26 weeks from the date of presenting their prescriptions at the pharmacy; cost effectiveness of the intervention versus current practice at 10 weeks and 26 weeks; in-depth qualitative understanding of the operationalization of NMS in pharmacies.

Secondary outcomes: impact of NMS on: patients’ understanding of their medicines, pharmacovigilance, interprofessional and patient-professional relationships and experiences of service users and stakeholders.

Economic analysis: Trial-based economic analysis (cost per extra adherent patient) and long-term modeling of costs and health effects (cost per quality-adjusted-life-year) will be conducted from the perspective of National Health Service (NHS) England, comparing NMS with current practice.

Qualitative analysis: a qualitative study of NMS implementation in different community settings, how organizational influences affect NMS delivery, patterns of NMS consultations and experiences of professionals and patients participating in NMS, and patients receiving current practice.

Sample size: 250 patients in each treatment arm would provide at least 80% power (two-tailed alpha of 0.05) to demonstrate a reduction in patient-reported non-adherence from 20% to 10% in the NMS arm compared with current practice, assuming a 20% drop-out rate.

**Discussion:**

At the time of submission of this article, 58 community pharmacies have been recruited and the interventions are being delivered. Analysis has not yet been undertaken.

**Trial registration:**

Current controlled trials: ISRCTN23560818

Clinical Trials US (clinicaltrials.gov): NCT01635361

## Background

Favorable outcomes in long-term conditions depend on self-management by patients, including appropriate medicines use. About 25% of medicines prescribed for long-term conditions are not taken as directed [1,2], and 15% of people receiving new medicines take few, if any, doses [3]. Many have problems with their medicines and have information needs, but often fail to discuss these concerns with their prescriber. Furthermore, prescribers do not ask about, and so are generally unaware of, patients’ behavior regarding following instructions, experimentation and self-medication with other therapies [4,5]. Prescribers may overestimate adherence [6], and be reluctant to voice suspicions about non-adherence [7]. Harm caused by non-adherence includes poor health-related quality of life, increased hospitalizations and premature mortality [8-11]. Wider burden includes cost to patients, healthcare providers and society. Improving adherence in asthma, type 2 diabetes and hypertension could save the English National Health Service (NHS England) £290 million, annually [12].

### Definition of adherence

In this study we have taken the definition of adherence from the World Health Organization: ‘the extent to which a person’s medication-taking behavior, following a diet, and/or executing lifestyle changes, corresponds with agreed recommendations from a healthcare provider’ [2]. In this study we operationalize adherence behavior by defining a patient as non-adherent if any scheduled doses were missed at various time points as a result of different adherence measures deployed.

### Pharmacist-provided interventions to improve medicines use

Pharmacist-provided interventions exist to facilitate improved medicines usage and health outcomes [13-26]. They are also viewed by some as a means to enhance pharmacist professional status [27]. Some interventions are effective [13-15,25,26], but not all [20,22-24]. There is little evidence around cost effectiveness of clinical community pharmacy interventions [28-30]. Most studies have methodological limitations: absence of a control, exclusion of pharmacist employment cost, use of intermediate outcomes, exclusion of health benefits, and absence of incremental analysis [28,31]. Interventions to improve medicines adherence have been disappointing in producing sustained behavior change [32], consistent health benefits, [33] or demonstrable cost effectiveness [31,34]. This can be attributed to poor study design, not using evidence on reasons for non-adherence, poor intervention development, lack of understanding of intervention complexity and its effects, and lack of integration into service delivery [35-37].

Implementing clinical services run by community pharmacists has been hampered by insufficient integration into patient pathways; poorly developed relationships between pharmacists and general practitioners (GPs); lack of access to patient information; inadequate methods for targeting services; and pharmacists’ lack of willingness to provide the service [38]. Medicines Use Reviews (MURs) are a recent example of a community pharmacy-based intervention. However, there has been variable uptake of MURs, mostly in chain pharmacies [39-41]. MURs have been carried out to variable standards by pharmacists, [42,43], partly due to variable understanding of what constitutes an MUR [44].

### The New Medicine Service

The New Medicine Service (NMS) [45] in England is based on evidence derived from our research that studied doctor-patient communication concerning medicines [46-49]. This and subsequent work established that problems with newly prescribed medicines appeared rapidly, were widespread and that a significant proportion of patients on a long-term medication quickly become non-adherent [50]. A pharmacist’s intervention could significantly reduce reported problems and non-adherence in a cost effective manner [51,52]. Within the NHS community pharmacy contract, the NMS is classified as an Advanced Service, whereby community pharmacists can opt to provide the service from their pharmacy [45]. This comes at a time when efficient medicines use could not be more important, in the face of economic pressures on the public sector budget. England’s ageing population now receives 50% more prescriptions items per capita for conditions such as heart disease, stroke, diabetes, COPD and asthma than in 1990 [53]. This proposed study is therefore timely as it is essential to evaluate whether NMS is effective, cost effective and acceptable to patients and healthcare providers. Supplementary policy-relevant outcomes from this service are opportunities to intervene regarding lifestyle and potential improved tracking of medicines-related adverse events by community pharmacists and patients [54].

### Aims of the study

The aims of this study are to:

•evaluate effectiveness and cost effectiveness of the NMS, from an NHS perspective, to inform decisions about continuation of the service

•explore operation of the NMS to determine acceptability to patients, pharmacists and GPs, indicators of successful implementation, generalizability and replicability across four therapeutic groupings in multiple pharmacy settings

### Specific objectives

This study has two work streams: an RCT and a qualitative investigation/appraisal. The purpose of the RCT is to evaluate effectiveness and cost effectiveness of the NMS from an NHS perspective to inform decisions about continuation of the service.

Data collected from the RCT will be used to:

•evaluate the impact of the NMS on patient medicines-taking behavior, patient outcomes, and cost effectiveness from an NHS perspective

•determine patients’ understanding of their medicines and the extent to which they are informed and supported in their medicines-related behavior

•examine whether NMS encourages pharmacovigilance by community pharmacists and patients

•inform and support future implementation and support development of outcome and quality measures for community pharmacy

The purpose of the qualitative workstream is to understand how NMS is implemented and experienced as a situated complex healthcare intervention. This involves investigation and analysis of:

•the implementation of NMS in different community pharmacy settings to determine the influence of local organizing factors

•the organization and configuration of NMS in different community pharmacies to determine variation in NMS operation and delivery

•the delivery of the NMS as a situated social practice, including interactions between multiple stakeholders (GP-pharmacist; pharmacist-patient; patient-pharmacist)

•the experiences and reflections of professionals and patients involved in the NMS

Evidence generated will allow better understanding of results obtained from both technology appraisal and NMS audit, through providing detailed case evidence of how and why the NMS is implemented in practice, including variations in implementation and uptake.

## Methods/design

### Trial design

This study will involve community pharmacies in England. It is a multi-center, pragmatic RCT involving a parallel group design. The health technology appraisal will assess the effectiveness and cost effectiveness of NMS via a patient-level pragmatic RCT of NMS versus current practice combined with modeling of long-term economic impact. Results generated from the technology appraisal will be combined with NMS audit data to provide wider estimates of effectiveness and cost effectiveness.

### Qualitative study design

The qualitative workstream comprises three distinct research activities:

a) pharmacy profiling will generate data on the organizational variables associated with the implementation, configuration and delivery NMS. Profiling will involve ethnographically informed observations and semi-structured interviews within participating community pharmacies, including non-participatory observations of work organization and pharmacy practice, guided tours, shadowing of pharmacy staff and interviews with pharmacy staff over a three to five day period. Observations will be guided by a standardized profiling tool to acquire common data set from across different sites.

b) patient tracking will generate data on the real-time delivery of NMS as a situated social interaction. Based upon pharmacy profiles, it involves producing a descriptive understanding of the ‘expected’ patient pathway to identify key interaction, communication and decision-making points followed by focused observations of, and interviews with, pharmacist and patient ‘before’, ‘during’ and ‘after’ the NMS consultation. A range of methods of recording data will be offered to participants, including video and audio recording or direct observation by research staff. Participant patients to be tracked will be recruited via the patient pool from the RCT.

c) stakeholder interviews will generate additional qualitative understanding on the organization and experience of NMS from the perspective of community pharmacy staff including pharmacists, dispensers and technicians. Patients who decline the invitation for an NMS in the pharmacy will also be invited to take part in short semi-structured interview to incorporate their views. Additionally, one-to-one interviews will be conducted with patients whose voices are seldom heard, and pharmacist and GP interviews will also be conducted to explore the professional perspective of the NMS.

### Eligibility for pharmacies to join the trial

Community pharmacies providing NMS in East Midlands and South Yorkshire (EMSY) and Greater London (GL) are eligible. Pharmacy selection will take into account known variables that influence organizational structures, workflow and integration, including:

•ownership: independent; small multiple; large multiple; supermarket

•proximity to GP: co-location; less than 500 meters; 500 meters to one km; over one km

•setting: urban; suburban; rural

•economic deprivation: based on Economic Deprivation Index

### Inclusion criteria

Community pharmacies will be eligible to take part in the RCT if they meet the following criteria:

•they provide care for patients starting a new medicine for one of four therapeutic groups: asthma/chronic obstructive pulmonary disease (COPD), type 2 diabetes, antiplatelets/anticoagulants or hypertension

•they are able to understand the participant study documents

•they are able and willing and able to provide consent

•they are accredited to provide the New Medicines Service

### Exclusion criteria

Community pharmacies will not be eligible to take part in the RCT if:

•they are outside of the EMSY and GL area

•they currently do not provide NMS or undertake an insufficient number to ensure recruitment to the study (< two per week).

•the pharmacy does not have a regular pharmacist (working on most days)

•the pharmacist is unable or unwilling to provide consent

### Eligibility for patients to join the trial

#### Patient inclusion criteria

The study will include:

•community dwelling patients eligible for the NMS (starting a new medicine for asthma/COPD, type 2 diabetes, antiplatelet/anticoagulation or hypertension)

•participants who are able to understand and consent to the NMS and also the study and who are willing to provide written consent/assent.

#### Patient exclusion criteria

•those not eligible for the NMS such as

•patients collecting a repeat prescription for a medicine (that is, not new)

•patients collecting a medicine where the only change from the previous medicine involves a dosage or formulation change only

•participants who are unable to understand patient/participant study documents

•participants who are unable and unwilling to provide written consent/assent

•those patients aged 13 and under

### Recruitment

#### Community pharmacies

Pharmacies will be recruited using a pragmatic convenience sample to enable a representative sample across the four eligibility criteria (ownership, proximity to the GP, setting, and economic deprivation). Local pharmaceutical committees and other regional and national pharmacy bodies in the GL and EMSY have been approached to assist in finding suitable research sites, as were the superintendent pharmacists of a range of multiple-owned pharmacy organizations.

#### Patients for RCT

The initial approach to participants will be from the community pharmacy that is providing the patient with NMS. Patients who have consented to the NMS will be invited to take part in the study. It will be explained to patients that taking part in the study is optional and if they decide not to take part they will be offered the NMS service as normal.

The study-designated pharmacist will inform the patient or their nominated representative (other individual or other body with appropriate jurisdiction), of all aspects pertaining to participation in the study. If the patient agrees to take part, the pharmacist will randomize the patient to receive the NMS or allocated to receive current practice. Patients could, if they were eligible for the NMS, volunteer for the study once they have seen the study poster displayed in the pharmacy. If a study-designated pharmacist is not present in the pharmacy at the time the patient presents with a prescription for a new medicine eligible for the NMS, the patient cannot be recruited into the study.

#### Participants for the qualitative analysis

##### Pharmacy profiles

Twenty pharmacies will be recruited for pharmacy profiling from those sites recruited to the RCT. These community pharmacies will be selected across geographical areas with the intention of investigating differences in pharmacy type, location, ownership, organization, and staffing.

##### Patient tracking

Participants will be identified and their NMS consultation recorded from a range of pharmacies and patient characteristics. Written informed consent will be taken before undertaking observation and interviews. The first two patients recruited into the study in each pharmacy will not be considered for tracking to allow the study pharmacist opportunity to familiarize themselves with the study paperwork.

Semi-structured interviews will be undertaken with community pharmacy and GP staff. A purposive sampling strategy will be used to identify and recruit a range of participants linked to the participating pharmacies: pharmacists, pharmacy technicians, counter assistants, GPs, nurse prescribers and patients in both geographical regions. Written informed consent will be obtained before interviews will take place.

### Interventions

#### Current practice

Those patients randomized to this arm will receive current practice where the patient receives their prescription, with advice as clinically necessary from the pharmacist at point of supply. It is not routine for patients to have any follow-up in this arm. Patients in this arm are not restricted from contacting the pharmacist for advice should they wish to.

#### NMS intervention

**Figure 1:**
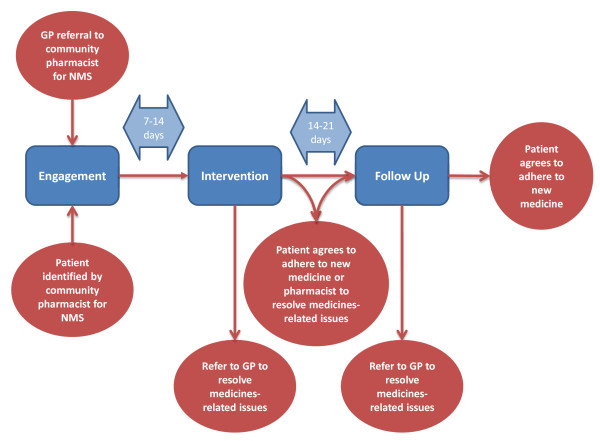
NMS intervention.

Those patients randomized to this arm will receive the NMS. The NMS will be offered by community pharmacists to people starting a new medicine for asthma/COPD, type 2 diabetes, hypertension or antiplatelet/anticoagulant treatment. The NMS can be summarized as patient engagement, intervention and follow-up (Figure 1). This is described in the NMS service specification [45].

### Study pharmacist training

All pharmacists who would be recruiting and consenting patients to be in the study will be required to attend a one day face-to-face training session at one of the host institutions. Training will be delivered by the study team and includes an overview of the study, study aims and objectives, research governance (including principles of Good Clinical Practice (GCP)), conducting the study in your pharmacy and interactive practice sessions.

Pharmacist study training will be followed up by an abridged training session in each pharmacy. This training provides all staff members with an overview of the study and enables them to assist the pharmacist in identifying potential study participants.

### Allocation of trial intervention

Following the consenting process, patients will be randomized into their respective study groups. The randomization sequence will be generated by the study statistician (RM).

Patients will be 1:1 randomized into one of the two study arms stratified by drug/disease group within each pharmacy using the SAS statistical software (version 9) (SAS UK Headquarters Wittington House Henley Road Medmenham Marlow, Buckinghamshire SL7 2 EB). Block randomization at the pharmacy level is necessary to avoid imbalances due to individual pharmacy variability. Separate randomization sequences were produced for patients 16 years and over and for patients aged 14 and 15 years old. The separation by age is required as study documents for the 14 and 15 year-old group include patient assent and parental consent instead of the usual normal patient consent forms. It is anticipated that there will be an unequal distribution across the four groups; therefore, stratification by drug/disease group is necessary, each with a different baseline adherence and/or effect size. The intervention arm will receive the NMS as per service specification while the control arm will receive care as per current practice. Concealment of sequence allocation will be achieved as pharmacists will randomly allocate patients to the two study arms using disease and age group specific, sequentially numbered tamper-proof opaque sealed envelopes containing details of allocation group. Periodic checks will be undertaken by a researcher to assess the integrity of study sites adhering to the randomization procedures. The sequence of treatment allocations will remain concealed until analysis is completed. Exceptions to this will include the need to reveal the randomization code because the patient has been identified as suitable for the qualitative work stream. As patients are registered into the study, checks will be conducted to ensure compliance with the registration protocol.

### Outcome measures

**Figure 2:**
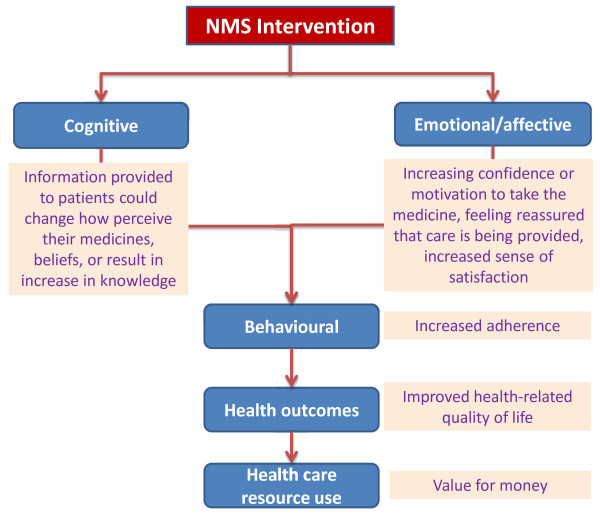
Intended outcomes of NMS intervention.

A successfully implemented NMS has five levels (see Figure 2) [55].

A summary of outcome measures, at which time points they are collected, and the method of collection is provided in Table 1.

**Table 1:** Summary of outcome measures

**Outcome measure**	**Time point recorded**	**Method of recording**
Health status	0 weeks	Questionnaire
EuroQol-5 dimension-3	6 weeks	
Level instrument	10 weeks	
(EQ-5D-3 L)	26 weeks	
Adherence	6 weeks	Questionnaire
Morisky’s medication	10 weeks	
Adherence scale 8-item version (MMAS-8)	26 weeks	
Adherence	6 weeks	Questionnaire
Visual analogs scale	10 weeks	
(VAS)	26 weeks	
Adherence	6 weeks	Telephone interview
NMS service question	10 weeks	
	26 weeks	
Medicines	6 weeks	Questionnaire
Understanding	10 weeks	
Beliefs About Medicines Questionnaire (BMQ)	26 weeks	
Consultation satisfaction	6 weeks	Questionnaire
Medical interview satisfaction scale-15		
(MISS-15)		
Health resource use	0 to 26 weeks inclusive	Self-completed diary

### Primary outcome measures

The primary endpoint for the RCT is to establish the impact of the NMS on patient medicines-taking behavior through measurement of self-reported adherence. The adherence measure in the interview schedule for the NMS intervention [56] and follow-up interview is a simple question: ‘People often miss taking doses of their medicines, for a wide range of reasons. Have you missed any doses of your new medicine, or changed when you take it? (Prompt: when did you last miss a dose?)’

Adherence in our original work was measured using self-report [57], using a similar question to that in the NMS interview. The patient was defined as non-adherent if any doses were missed in the previous seven days, partly because simply asking patients about their adherence will identify at least 50% of those with low adherence, with a specificity of 87% [58]. We will validate the NMS adherence measure by using an existing validated scale alongside it (Morisky Eight Item Medication Adherence Scale (MMAS-8)) [59].

### Secondary outcome measures

We are collecting data on a number of secondary outcome measures relating to number of medicines-related problems reported by patients, and resolved; adverse events and NHS contacts associated with adverse events; health status; resource use and costs; length of initial and subsequent NMS consultations; NHS contact (primary and secondary care) and non-medical costs.

### Composite outcomes

In the original work, most ‘withdrawals’ were that the patients in the intervention arm were referred back to the GP by the pharmacist due to side effects, lack of effect or patient non-adherence’ [51]. In this study, these events are being recorded and will be combined into a composite outcome.

Adherence will be reported as adherence in the group eligible to be adherent (that is, still meant to be taking the medicine). We will also report patients referred back to the GP (whether a new medicine is prescribed or not) as a separate outcome. This information will be available from the six week call for both intervention and current practice arm.

A composite outcome that combines proportion of adherent patients and patients appropriately referred back to the GP will be derived and summary statistics presented.

### Data for the economic analysis

#### Costs of the intervention

Costs will be incurred at the patient level, in delivering the intervention. Variable costs refer to items where the quantity of resources used is determined only by the need for them as inputs to individual patient care. Variable resource use associated with the interventions (time spent, costs of telephone calls, printing and posting) will be recorded for each patient. Fixed costs are those costs that are not affected by patient activity in the short term. UK standard costs will be used for unit costs. This may somewhat over- or under-estimate local unit costs, but allows explicit comparison of costs and local adjustments can be made. Unit costs associated with the intervention will be obtained from the Personal Social Services Research Unit (PSSRU) [60], Department of Health reference tables and other reference costs.

#### Clinical and economic impact of non-adherence

The trial will not observe patient outcomes and NHS costs resulting from non-adherence to their newly prescribed medicine. Rather, outcomes will be derived from published evidence on the link between adherence improvement and impact on health. The NMS study is not designed to calculate the impact of the intervention on patient health outcomes, either in terms of sample size or length of follow-up. Use of proxy measures such as number of primary and secondary care contacts (hospital admissions, accident and emergency visits and outpatient visits) may be subject to difficulties if considered as patient outcomes. This is because the intervention may lead to increased NHS contact in the short term. Thus, we will estimate the long-term effect of the observed adherence improvements on patient outcomes and NHS costs. Data on natural history of diseases, treatment effectiveness, resource use, and health status (utility) will be obtained from published literature to populate the model.

**Figure 3:**
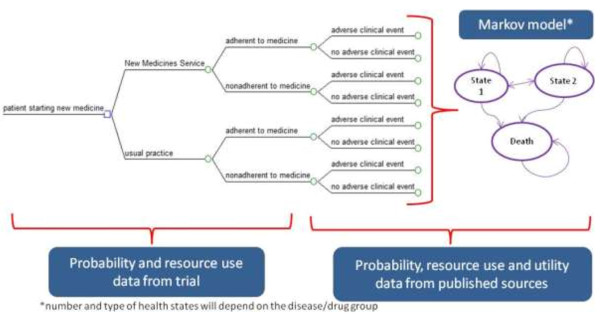
Decision-analytic model for NMS economic evaluation.

We will develop at least one treatment pathway model for each of the four treatment groups targeted by NMS, encompassing the consequences of being adherent or non-adherent (see Figure 3). A common generic approach will be used to develop the models. We will undertake the economic analysis from the perspective of the English NHS in terms of the direct costs of providing an intervention to improve medicines adherence in chronic illnesses and the long-term costs of managing the conditions for adherent and non-adherent patients (to estimate cost of non-adherence).

Markov models will be designed and populated, incorporating the measures of uncertainty around the point estimates, to conduct probabilistic sensitivity analysis. The UK Treasury recommended 3.5% discount rate for both costs and outcomes will be used.

Literature review will be used to obtain published utility weights to allow quality adjusted like year (QALY) generation and cost utility analysis. The baseline EuroQol 5D-3 L (EQ-5D) in our trial cohort will be incorporated into the QALY generation.

We will model the effect of the observed adherence improvements on patient outcomes and NHS costs. Probability, resource use and health status (utility) data will be obtained from published literature to populate the model.

### Data for the qualitative study

It is anticipated that data for the qualitative study will be obtained from 20 pharmacy profiles, 12 patient tracking, 24 patient interviews (12 for each NMS and current practice arm), 24 pharmacist interviews and eight GP interviews. Interviews will incorporate a range of patient and professional views and will be digitally recorded and transcribed. All accompanying field notes will be retained.

### Adverse events

The nature of the study presents a low risk to participants. In the unlikely event of a suspected adverse event, these will be reported to the study principal investigator and appropriate action taken on a case-by-case basis. All adverse events will be reported to the study sponsor.

### Sample size

The estimated change in prevalence of non-adherence behavior (primary outcome) is expected to fall from 20% to 10%. A sample size of 250 patients/arm is sufficient to detect this change with 80% power, 5% significant level (two-tailed) at a projected 20% drop-out rate. Therefore, the total number of patients to be recruited for the RCT is 500 patients.

Data obtained a few months post-NMS implementation indicated that on average, two NMS consultations were initiated per pharmacy per week. It was initially estimated that on average there will be 52 eligible patients per pharmacy in six months. Based on the assumption that 50% of eligible NMS patients consent to be part of the study, there will be 26 in each pharmacy over the required six months.

### Revision of clinical site recruitment strategy

To deliver the sample as quickly as possible, patients were to be recruited from 20 pharmacies which, assuming the above drop-out rate, would deliver 520 patients within six months. However, of 24 pharmacies initially recruited, at least ten have not delivered patients to the study due to lack of both NMS service uptake and patients declining to be in the study. Therefore, we are currently replacing these pharmacies with ‘NMS-active’ pharmacies. The pharmacy profiling has provided insights into the reasons that affect service uptake and facilitators to recruitment to the study. This has enabled us to develop a site suitability survey to better identify more appropriate and potentially more successful pharmacy sites for recruitment (see Additional file 1: Table S1).

### Compliance

We recognize that it can be a challenge to encourage community pharmacies and patients using community pharmacies to engage in interventions. We believe that the risks of non-compliance will be minimized by providing pharmacies with clear information on what the study involves, providing access to members of the research team to answer queries and address problems experienced by the pharmacies.

### Likely rate of loss to follow-up

In the original work, 20% patients withdrew from the study due to a range of reasons [51]. The main reason was that the patients in the intervention arm were referred back to the GP by the pharmacist due to side effects, lack of effect or patient non-adherence. In this study, these events are being recorded and will be combined into a composite outcome. Loss to follow-up for other reasons is expected to be less than 10%.

### Withdrawal of patients from the study

A patient will be considered to have withdrawn from the study if the study team receives any notification that the patient wishes to withdraw. This notification might come from the patient themselves, the patient’s representative or from the patient’s pharmacist. We have accounted for withdrawal in our sample size calculations to minimize the effect on the analysis.

As the NMS is a nationally available service which all patients have the right to receive, some patients allocated to the current practice study arm may decide post-randomization that they still wish to receive the NMS. Should this occur this will be counted as a withdrawal at randomization. If a patient ‘loops’ through the NMS due to referral back to their GP or addition/change of medication (which may mean they received a second new medicine so will start at the beginning of the NMS for this medicine), this patient will continue in the RCT/study as normal. The interaction with the GP and/or change in medication will be noted by the researchers.

### Patient/public involvement

AC is co-investigator on the project and is an equal member of the team. By attending monthly meetings in person, he has been able to challenge, input and advise the project at every step, bringing his experience and expertise of both the world outside academia and his time living with a long-term condition, to the work. AC was also involved in delivering training to the NMS study pharmacists and in organizing the stakeholder day. We have had further input from a group of patient representatives in development of:

•the NMS evaluation study application submitted to the DOH

•the content of materials used for patient recruitment (mainly information sheets and consent forms)

•data collection forms used during the follow-up telephone calls

•the diary for collecting health care resource use data

•the presentation used for the NMS study pharmacist training day

•training of the NMS study pharmacist

•frequently asked questions for the study website

We have a website that provides information to interested parties. (http://www.nottingham.ac.uk/).

### Statistical analysis

#### Descriptive analyses

Continuous data will be explored using means and standard deviations (SD) if approximately normally distributed and medians and inter-quartile ranges (IQR) if non-normally distributed. Categorical data will be described using frequencies and percentages.

#### Comparing baseline characteristics between treatment arms

The following characteristics will be described by treatment arm: patient age, gender geographic location, disease group and diagnosed person years; (Additional file 1: Table S2).

#### Comparisons between treatment arms

An intention-to-treat analysis will be used such that patients will be analyzed in the arms they were allocated to, regardless of whether they received the intervention or not [61,62].

Data analysis will be conducted by the research team and by the study statistician (RM). To compare adherence rates, differences in categorical variables will be analyzed using the chi-squared test or Fisher’s exact test as appropriate. The relationship between non-adherence and treatment group will be investigated using logistic regression models to adjust for interaction with chronic disease and other potential confounders. The significance of the variables in the model will be assessed using the Wald chi-squared test and determination of odds ratios (ORs) with associated 95% confidence intervals (CIs). Goodness of fit to the model will be assessed using the Hosmer and Lemeshow test.

Data collected from the study will be compared with anonymized data from available NMS national data sets. This will facilitate generalizability of the findings.

Statistical significance will be assessed at the 5% (two-sided) level. All statistical analyses will be conducted using SPSS16 (SPSS IBM United Kingdom Limited, PO Box 41, North Harbour, Portsmouth, Hampshire, PO6 3 AU)and STATA 11.0 (STATA: Timberlake Consultants Limited B3 Broomsleigh Business Park Worsley Bridge Road, London SE26 5BN United Kingdom) [63].

### Primary outcome

The primary outcomes will report on the impact of the intervention on adherence to prescribed medicines.

If any patients are lost to follow-up, a sensitivity analysis will be undertaken [61,62].

### Secondary outcome measures

We acknowledge the potential for type 1 errors associated with significance testing for multiple end points. We will therefore consider our analyses of secondary outcome measures to be partly exploratory in nature, and partly confirmatory of our findings for the primary outcome measures.

•process measures: parameters from NMS audit data set

•cognitive and behavioral outcome (self-reported adherence (NMS question and MMAS-8))

•number of medicines-related problems reported by patients, and resolved

•adverse events and NHS contacts associated with adverse events

•health status (EQ-5D)

•resource use and costs

•length of initial and subsequent NMS consultations

•NHS contact (primary and secondary care)

•non-medical costs

### Within trial analysis of costs

Costs calculated in the trial analysis will be the cost of intervention for each patient enrolled in the trial for both treatment arms. These data will be presented separately for the two treatment arms. Comparisons between treatment arms at patient level will be made using a two-sample *t*-test on the original dataset, or on a bootstrapped dataset, depending on the normality of the distribution of costs [64].

### Sub-group analyses

Sub-group analyses [65] will only be undertaken for primary outcome measures. Analyses will be undertaken to assess whether the effect of the intervention varies by disease type, age, gender, pharmacy type, pharmacy location, time since diagnosis, number of other medicines prescribed, and deprivation index. Treatment arm and the (continuous) covariate of interest will be added into the regression model [65]. Where there is evidence of non-linearity, this will be investigated and appropriate transformations will be performed. Significance will be assessed based on likelihood ratio tests with a *P* value of < 0.05 taken as significant and *P* values between 0.05 and 0.1 described as there being ‘some evidence’ for an interaction.

### Missing data

A complete case analysis will be undertaken with a range of approaches for undertaking sensitivity analyses to assess the robustness of the findings with respect to missing data.

### Economic analysis

A standard approach to economic analysis will be applied [66]. We propose to undertake the economic analyses from the perspective of the NHS in terms of the direct costs of providing an intervention to improve medicines adherence in primary care, and the costs of managing diseases for adherent and non-adherent patients.

### Comparators and key parameters under investigation

The evaluation will compare the NMS intervention with current practice. The main outcome of the economic analysis is cost per QALY gained. We will examine the differences in overall NHS costs and in QALYs gained between NMS intervention and current practice patient groups. Additionally, trial-based economic analysis will be conducted and cost per extra adherent patient will be estimated.

### Sample size for the economic analysis

The study cannot be powered to detect differences in costs because there is no prior study upon which to base a power calculation.

### Time horizon (follow-up period)

Adherence rates in both groups will be followed up for six months following the completion of the intervention. The Markov models will follow up patients for long-term horizon (to capture all the relevant cost and outcome consequences for each disease group). Life time horizon will be considered.

### Modeling the effect of non-adherence in each disease group

Each disease-specific Markov model will be populated with transition probability, cost and health status data to generate the outcomes and costs in a cohort of adherent patients and in a cohort of non-adherent patients.

### Estimating the cost effectiveness of the NMS intervention

#### Trial-based cost-effectiveness analysis (short-term economic evaluation)

Trial-based economic evaluation will be conducted. The adherence measures, intervention costs, and total costs incurred during the trial horizon will be generated at patient level. Then, the adherence rates in the NMS and current practice arms and cost per extra adherent patient will be calculated. Both deterministic and probabilistic incremental economic analyses will be carried out using the adjusted cost and outcome data, in combination with the NMS intervention costs.

The incremental cost per extra adherent patient generated by the NMS intervention over current practice will be calculated using the following equation:

$$\begin{array}{l} \mathrm{ICER}=\left({\mathrm{Cost}}_{\mathrm{NMS}}–{\mathrm{Cost}}_{\mathrm{Current}\;\mathrm{practice}}\right)/\\ \;\left(r_{\mathrm{NMS}}–r_{\mathrm{Current}\;\mathrm{practice}}\right), \end{array}$$

where r_NMS_, r_Current practice_ is the proportion of adherent patients in the NMS arm, and current practice arm, respectively. Statistical analysis is not appropriate to test the robustness of ICER. It is not possible to generate 95% confidence intervals around ICERs because the ratio of two distributions does not necessarily have a finite mean, or therefore, a finite variance [67]. Therefore, generation of a bootstrap estimate of the ICER sampling distribution to identify the magnitude of uncertainty around the ICERs is required. Bootstrapping with replacement will be employed, utilizing Microsoft Excel (Microsoft Campus, Thames Valley Park, Reading, Berkshire, RG6 1WG), using a minimum of 5,000 iterations. These incremental costs and outcomes will be plotted on cost effectiveness plane, uncertainty around ICER will be investigated and cost effectiveness acceptability curves (CEACs) [68,69] will be constructed.

#### Cost-utility analysis (long-term economic evaluation)

Combining the NMS trial results with the disease-specific models will allow us to estimate the costs and outcomes associated with the NMS intervention versus current practice.

The adherence rates for each disease group occurring in the NMS and current practice arms will be combined with the disease-specific Markov models (see Figure 3). The incremental costs and outcomes associated with each disease group will be estimated based on trial combined with economic model. This will allow us to generate the incremental effect of the NMS intervention on the costs and outcomes for each disease group, and overall (for the population for which NMS service is targeted).

Incremental economic analyses will be carried out using the adjusted cost and outcome data (observed in the trial) in combination with the NMS intervention costs, and long-term costs and health effects (estimated using Markov models). This will generate the estimates of the overall costs and health outcomes, measured by QALY gained, for the NMS and the current practice arms.

The incremental cost per extra QALY generated by the NMS intervention over current practice will be calculated using the following equation:

$$\begin{array}{l} \mathrm{ICER}=\left({\mathrm{Cost}}_{\mathrm{NMS}}–{\mathrm{Cost}}_{\mathrm{Current}\;\mathrm{practice}}\right)/\\ \;\left({\mathrm{QALY}}_{\mathrm{NMS}}–{\mathrm{QALY}}_{\mathrm{Current}\;\mathrm{practice}}\right). \end{array}$$

#### Sensitivity and scenario analysis

Sensitivity analysis is required to assess the level of uncertainty in the data collected within the trial and subsequent internal robustness of the results.

Several deterministic one-way sensitivity analyses will be conducted (testing the impact of key uncertain parameters on the cost-utility result). Scenario analyses will be proposed and discussed, alternative key assumptions will be tested.

Probabilistic sensitivity analysis (PSA) will be conducted for base-case scenario, main alternative scenarios (and one-way sensitivity analyses).

Monte Carlo simulation will be applied for sampling incremental costs and QALY, using Tree Age Pro (TreeAge Software, Inc., 888-TreeAge -or- +1 413-458-0104, One Bank Street Williamstown, MA, 01267 USA) (at least 1,000 samples). Uncertainty around input parameters will be modeled in a standard way: appropriate probability distributions will be assumed for cost, utility, probabilities and ratios [70].

Cost effectiveness acceptability curves (CEACs) [68] will be constructed to express the probability that the cost per QALY gained (y-axis) is cost effective as a function of the decision-maker’s ceiling cost effectiveness ratio (λ) (x-axis) for base-case, sensitivity and scenario analyses [69].

The incremental net monetary benefit (INB) will be estimated from the incremental costs and QALYS for NMS compared with current practice using the formula:

$$\begin{array}{l} \mathrm{INB}\left(\lambda \right)=\lambda \left({\mathrm{QALY}}_{\mathrm{NMS}}–{\mathrm{QALY}}_{\mathrm{Current}\;\mathrm{practice}}\right)\\ \;-\left({\mathrm{Cost}}_{\mathrm{NMS}}–{\mathrm{Cost}}_{\mathrm{Current}\;\mathrm{practice}}\right) \end{array}$$

The incremental net benefit approach will be used due to well-known problems associated with incremental cost effectiveness ratios (ICERs) when bootstrap replicates cover all four quadrants of the cost effectiveness plane [71,72]. Incremental net monetary benefit will be calculated for a threshold range from £0 to £160,000 using increments of £10,000.

### Format of tables for publishing the main trial results and within trial economic analysis

The format of tables for publishing the main trial results and economic analysis is shown in Additional file 1: Table S2.

### Qualitative study

Qualitative data analysis will start during the early stages of data collection and proceed iteratively so that emergent findings are incorporated into subsequent data collection, including the revision of data collection methods, such as interview topic guides. All pharmacy profile observations will be recorded in field notes and subsequently typed up. All interviews will be transcribed verbatim. The data will be then imported into qualitative analysis package NVivo; QSR International Pty Ltd. Version 9, 2010 (QSR International (UK) Limited, Warrington, Cheshire, WA2 7LT, United Kingdom) for the purpose of coding and thematic analysis. This will involve initial reading and re-reading of the transcribed data by multiple members of the research team to identify common codes and categories. These codes will be compared for their internal consistency and boundaries. A coding framework will be constructed iteratively and two members of the research team will systematically code data according to this framework. Coded extracts will be compared and differences of opinion discussed until agreement is reached. Actively searching for disconfirming data will be undertaken as well as regular detailed discussions amongst the qualitative researchers. To enhance the consistency of analysis, review meetings will be held with a third researcher (JW) who will oversee the process and negotiate consensus on the final thematic codes assigned to each response.

Consideration will then be given to how these issues group together in broader themes related to the research objectives. The principle of constant comparison will be used to test and refine the empirical conceptual consistency of codes and themes which were synthesized and narrated using a technique similar to that used by Ziebland and McPherson (2006) [73].

### Trial organization

Professor Elliott and Dr Boyd will have overall responsibility for the day-to-day management of the trial and Professor Waring will have overall responsibility for the qualitative workstream. Professor Elliott will have responsibility for the economic analysis. Mr Mehta is the trial statistician.

A Project Management Group will be meeting monthly throughout the study to help ensure that all trial activities are organized according to the protocol and within the timescales set out in the original application for funding, will monitor and supervise the trial and comment on any proposed amendments to the protocol.

The NMS Evaluation Advisory Group (NEAG) is headed by Professor Nick Mays. The trial statistician will report to the NEAG, which will be responsible for reviewing the data from the trial. The NEAG has agreed to operate within the framework suggested in the *MRC Guidelines for Good Clinical Practice in Clinical Trials*[74].

### Ethical aspects

The clinical trial will be conducted according to the Helsinki Declaration [70], the GCP Guidelines [75] and NHS Research Governance requirements. Patients agreeing to participate in the study have provided written informed consent in a form designed for such purpose. The patient may refuse to continue participating in the study at any time after providing his/her consent. The information generated by the study will be confidential and limited to the purposes stipulated in the protocol.

The study was given a favorable opinion on 2 May 2012 by the National Research Ethics Service (NRES) Black Country Research Ethics Committee (12/WM/0096).

All staff involved in data collection will have approval from the appropriate local NHS research and development offices.

The study was registered with the ClinicalTrials.gov trials database on 19 June 2012. Trial reference number NCT01635361 (http://clinicaltrials.gov/ct2/show/NCT01635361).

The study was registered with the Current Controlled trials database on 5 July 2012. Trial reference number ISRCTN 23560818 (http://www.controlled-trials.com/ISRCTN23560818/; DOI 10.1186/ISRCTN23560818).

This study is registered with the UK Clinical Research Network (UKCRN) study 12494 (http://public.ukcrn.org.uk/Search/StudyDetail.aspx?StudyID=12494).

### Study timeline

Trial start: January 2012

Start of baseline data collection and interventions in pharmacies: August 2012

End of interventions in pharmacies: September 2013

End of six month follow-up data collection: March 2014 (one month after official funding ends)

Start of data analysis: September 2013

Planned study end date: February 2014

Duration: 26 months

## Discussion

As the NMS intervention is an advanced service being delivered by appropriately qualified pharmacists, we did not expect non-compliance with the intervention to be a large problem. However, some pharmacies were only offering the service to a limited number of patients, which has affected our patient recruitment rate and has required further site recruitment. We have also revised recruitment targets for individual pharmacies and developed a site suitability survey. On-going support provided to sites includes regular visits to sites have been made by members of the research team. Sites have also been kept up-to-date with recruitment progress through newsletters.

One strength of this study is the substantial qualitative workstream, supplemented by engagement and implementation activity. Many studies of pharmacy interventions have not included qualitative work. Qualitative work will enable us to learn about how this service is actually being implemented in practice and will enable us to explain the quantitative results we obtain. We will be carrying out stakeholder days where we will be inviting patients, service providers and commissioners to attend, to obtain views on the NMS in particular, and on managing medicines for long-term conditions in general.

## Trial status

At the time of submission of this article, 58 pharmacies have been recruited into the study. Seventy pharmacists have been trained and 502 patients have been recruited (4 October 2013). At the time of submitting this protocol, analysis of quantitative data had not been undertaken.

## Abbreviations

ACE: Angiotensin converting enzyme (inhibitor); CEACs: Cost effectiveness acceptability curves; CHD: Coronary heart disease; COPD: Chronic obstructive pulmonary disease; EMSY: East Midlands and South Yorkshire; EQ-5D-3 L: EuroQol 5 Dimension-3 level instrument; GL: Greater London; GCP: Good Clinical Practice; GP: General practitioner (or family practitioner); ICER: Incremental cost effectiveness ratios; INR: International normalized ratio; IT: Information technology; MMAS-8: Morisky’s medication adherence scale 8-item version; MRC: Medical Research Council; MUR: Medicine use review; NHS: The National Health Service in England; NMS: New medicines service; PCRN: Primary care research network; PCT: Primary care trust; PSSRU: Personal Social Services Research Unit; QALY: Quality-adjusted life-year; RCT: Randomized controlled trial.

## Competing interests

The authors declare that they have no competing interests.

## Authors’ contributions

RAE, who has made substantial contributions to the conception and design of the study, is co-responsible with MB for the overall administration and direction of the project, the analysis and interpretation of data and will give the final approval of the version to be published. MB, JW, NB, AC, RM and AJA are also co-responsible for the overall design, administration and direction of the study. NS, AL and JD are responsible for the day-to-day management of the trial and qualitative studies LT for economic modeling. All authors participated in the design of the project. RM and RAE have had a major role in designing the statistical analysis for the trial. RE and LT have designed the economic analysis; JW, AL, JD led on the design of the qualitative analysis. AC is the patient and public involvement lead, providing PPI perspective on all aspects of the study design, conduct and analysis and provides contacts for further patient input. All authors read and approved the final manuscript.

## Supplementary Material

Additional file 1 **File name: Elliott Additional file 1.** File format: MS Word (.doc). Title of data: site suitability survey and outline of results tables. Description of data: two tables. Click here for file
