# Supplementary material for: Protocol for the New Medicine Service Study: a randomized controlled trial and economic evaluation with qualitative appraisal comparing the effectiveness and cost effectiveness of the New Medicine Service in community pharmacies in England
Source: Trials. 2013 Dec 1;14:411. doi: 10.1186/1745-6215-14-411 (PMC4220816; doi:10.1186/1745-6215-14-411)
Supplement: Additional file 1 — File name: Elliott Additional file 1. File format: MS Word (.doc). Title of data: site suitability survey and outline of results tables. Description of data: two tables. [file 1745-6215-14-411-S1.doc]

**Additional file 1:**

**Table S1. Site suitability survey**

| Criteria | Score |
| --- | --- |
| Pharmacist’s enthusiasm – does the pharmacist see the purpose of doing NMS? Is the pharmacist motivated to incorporate this service into work routine? | ….../2 |
| Pharmacist’s skills and experience – do you think the pharmacist is a good communicator? Able and confident to ‘sell’ NMS to patients? (probe: observe how pharmacist introduce the service to patients) | …../2 |
| Organisation and time management – how much NMS is being routinised into pharmacist’s daily tasks? Is there efficiency i.e. clear and straightforward procedures to make an appointment? (probe: does the patients have to wait long before seeing a pharmacist for NMS? is there reminder system in place?) | …../2 |
| Staffing – is there second cover pharmacist or a locum pharmacist (to release one of them to do NMS/other extended services like MUR)? | …../1 |
| Volume of NMS – are there high volumes of NMS coming through the door? | …../1 |
| Volume – is NMS offered every day? (score 0 if only certain day(s) in a week NMS is offered) | …../1 |
| Clientele – regular clientele? (score 0 if turnover is high or mostly medications for acute conditions) | …../1 |
| Clientele – mostly comprise of traditionalists? (or patients who are more likely to receive NMS, e.g. educated patients) | …../1 |
| Environment – is the consultation room used regularly? (score 0 if consultation room looks congested, or being used as a store room) | …../1 |
| *Total* | …../12 |

***Table S2. Format of tables for publishing the main trial results and within trial economic analysis***

|  |  | ***Control Arm***  ***(Current Practice)*** | ***Intervention Arm***  ***(New Medicine Service)*** |
| --- | --- | --- | --- |
| ***n*** |  |  |  |
| ***Age*** |  | ***Mean ± SD (Range)*** |  |
| ***Gender*** | ***M*** | ***n*** |  |
|  | ***F*** | ***N*** |  |
| ***Location*** |  |  |  |
| ***Employment status*** | ***As per Questionnaire*** |  |  |
| ***Ethnicity*** | ***As per Questionnaire*** |  |  |
| ***Disease Group*** | ***Asthma/COPD*** |  |  |
|  | ***Hypertension*** |  |  |
|  | ***Type 2 diabetes*** |  |  |
|  | ***Antiplatelet/Anti-coagulant*** |  |  |
| ***New medicines at study entry*** | ***1*** | ***N*** | ***N*** |
|  | ***2*** | ***N*** | ***N*** |
|  | ***3*** | ***N*** | ***N*** |
|  | ***n…..*** |  |  |
| ***Diagnosis person years*** |  | ***Mean ± SD (Range)*** |  |
| ***Co-morbidities*** |  | ***Mean ± SD (Range)*** |  |
| ***Concomitant medicines*** |  | ***Mean ± SD (Range)*** |  |
|  |  |  |  |
|  |  |  |  |
|  |  |  |  |

***Results:***

***Outcomes no. at each time point, no of referrals (new medicines prescribed etc. if outcomes known***

***By disease state***
